# Blood-based inflammatory biomarkers in ovarian cancer: addressing the challenge of early detection, metastasis, and prognostic stratification

**DOI:** 10.3389/or.2026.1914468

**Published:** 2026-10-07

**Authors:** Emmanuel Ifeanyi Obeagu

**Affiliations:** 1 Division of Haematology, Department of Biomedical and Laboratory Science, Africa University, Mutare, Zimbabwe; 2 Department of Molecular Medicine and Haematology, School of Pathology, Faculty of Health Sciences, University of the Witwatersrand, Johannesburg, South Africa

**Keywords:** diagnosis, inflammatory biomarkers, metastasis, ovarian cancer, prognosis

## Abstract

Ovarian cancer remains a major cause of gynaecological cancer mortality, largely because early disease is frequently asymptomatic and reliable population-based screening remains unavailable. Systemic inflammation is increasingly recognised as an important component of ovarian cancer biology, linking tumour progression, immune dysregulation, angiogenesis, metastasis and treatment resistance. This narrative review critically evaluates blood-based inflammatory biomarkers, including C-reactive protein, interleukin-6, interleukin-8, tumour necrosis factor-α, fibrinogen, neutrophil-to-lymphocyte ratio, platelet-to-lymphocyte ratio, lymphocyte-to-monocyte ratio, systemic immune-inflammation index (SII), systemic inflammation response index (SIRI) and pan-immune-inflammation value (PIV), across early detection, metastatic assessment, treatment decision-making and prognostic stratification. Available evidence indicates that elevated inflammatory indices are consistently associated with advanced disease and adverse outcomes, although substantial heterogeneity exists in biomarker thresholds, populations, timing of measurement and analytical approaches. Recent evidence also suggests that preoperative NLR and SII may contribute to treatment selection, including neoadjuvant chemotherapy, although prognostic independence is not consistently demonstrated. Importantly, inflammatory biomarkers lack sufficient specificity for standalone ovarian cancer screening and should not replace CA-125, HE4, imaging or histopathological assessment. Their greatest clinical potential lies in multimarker models integrating systemic inflammatory, tumour-specific, molecular and clinical variables. Future research should prioritise prospective multicentre validation, standardised thresholds, longitudinal assessment and clinically interpretable prediction models to determine whether inflammatory profiling can advance precision risk stratification and treatment selection in ovarian cancer.

## Introduction

Ovarian cancer remains one of the most clinically challenging gynaecological malignancies and a major cause of cancer-related mortality among women worldwide. Its poor outcomes are driven largely by late diagnosis, aggressive tumour biology, extensive intraperitoneal dissemination and the development of treatment resistance. Early-stage ovarian cancer may produce vague or nonspecific symptoms, while clinically apparent disease is frequently already associated with peritoneal involvement, ascites, omental disease or metastatic spread. Despite advances in imaging, surgery, systemic therapy and molecular classification, a sufficiently sensitive and specific blood-based strategy for population-level early detection has not been established (1–3). CA-125 remains the most widely used circulating biomarker in ovarian cancer, while human epididymis protein 4 (HE4) provides complementary information and is incorporated into selected diagnostic algorithms. However, neither biomarker is sufficiently specific or sensitive to function independently as a population-screening test. CA-125 may be elevated in several benign gynaecological and inflammatory conditions, whereas its sensitivity is limited in some early-stage and non-serous tumours. These limitations have stimulated investigation of additional circulating biomarkers capable of capturing biological processes that accompany tumour initiation and progression (4–6).

Inflammation represents one such process. Ovarian cancer develops within a complex tumour microenvironment comprising malignant cells, mesothelial cells, fibroblasts, endothelial cells, macrophages, neutrophils, lymphocytes and platelets. Persistent communication among these cellular populations generates cytokines, chemokines, acute-phase proteins and coagulation mediators that can influence tumour growth and host immunity. Interleukin-6 (IL-6), interleukin-8 (IL-8), tumour necrosis factor-α (TNF-α), C-reactive protein (CRP) and fibrinogen have consequently attracted attention as circulating indicators of tumour-associated inflammation (7, 8). Systemic inflammation is not merely an epiphenomenon of advanced cancer. Inflammatory signalling may actively contribute to ovarian cancer progression through activation of pathways such as nuclear factor-κB and Janus kinase/signal transducer and activator of transcription 3 (JAK/STAT3), promotion of angiogenesis, epithelial–mesenchymal transition, immune suppression, tumour-cell survival and metastatic implantation. Neutrophils, monocytes and platelets may further support tumour progression, whereas lymphopenia can reflect impaired antitumour immune surveillance. These interactions have provided the biological rationale for readily available haematological indices, particularly the neutrophil-to-lymphocyte ratio (NLR), platelet-to-lymphocyte ratio (PLR), lymphocyte-to-monocyte ratio (LMR), systemic immune-inflammation index (SII), systemic inflammation response index (SIRI) and pan-immune-inflammation value (PIV) (9–11).

The growing literature, however, requires careful interpretation. Associations between elevated inflammatory biomarkers and advanced ovarian cancer or poor survival are frequently reported, but the evidence is characterised by substantial heterogeneity. Studies differ in patient populations, histological subtypes, disease stage, timing of blood sampling, treatment exposure, inflammatory cut-offs and statistical methodology. Furthermore, inflammatory indices are intrinsically nonspecific and can be altered by infection, autoimmune disease, obesity, medications and other comorbid conditions. Consequently, an association with ovarian cancer does not establish diagnostic specificity, prognostic independence or clinical utility. The relatively strong observational evidence for some inflammatory indices should therefore not be confused with evidence demonstrating that biomarker-guided management improves patient outcomes (12, 13). Recent clinical evidence also suggests that the potential application of inflammatory biomarkers may extend beyond prognosis. For example, Yalcin et al. reported that preoperative NLR and SII were independently associated with selection of neoadjuvant chemotherapy in patients with advanced high-grade serous ovarian cancer, although neither marker independently predicted survival. This finding is clinically important because it illustrates a potential role for inflammatory biomarkers in treatment decision-making, while simultaneously demonstrating why prognostic associations must be distinguished from actionable clinical utility (14).

Accordingly, the most plausible future role of blood-based inflammatory biomarkers is unlikely to be as standalone screening tests. Rather, they may provide complementary biological information when integrated with CA-125, HE4, imaging, clinicopathological characteristics and emerging molecular liquid-biopsy markers. Composite inflammatory indices may offer additional information regarding the interaction between systemic inflammation, coagulation and immune competence, but whether they consistently outperform simpler indices remains uncertain (15). This narrative review therefore critically examines the biological basis and clinical evidence surrounding blood-based inflammatory biomarkers in ovarian cancer, with particular emphasis on early detection, metastatic risk, treatment decision-making and prognostic stratification. Rather than simply cataloguing reported associations, the review evaluates the consistency, limitations and translational relevance of individual and composite biomarkers and considers how they may be integrated into multimodal precision approaches. It further identifies key evidence gaps and proposes priorities for prospective validation, biomarker standardisation and clinically meaningful implementation.

## Biological basis of inflammation in ovarian cancer

Inflammation is a fundamental component of ovarian cancer biology and represents a dynamic interaction between malignant cells and the host immune, stromal, vascular and coagulation systems. Rather than being simply a consequence of advanced disease, persistent inflammatory signalling can actively promote tumour initiation, progression, invasion, metastatic dissemination and therapeutic resistance. This tumour–host interaction provides the biological rationale for investigating circulating inflammatory biomarkers as indicators of disease activity and prognosis. However, because systemic inflammation is not specific to malignancy, its clinical interpretation requires integration with tumour-specific and clinicopathological information (16, 17). Ovarian tumours develop within a complex microenvironment containing tumour-associated macrophages, neutrophils, lymphocytes, dendritic cells, fibroblasts, endothelial cells and mesothelial cells. Malignant and stromal cells release cytokines, chemokines, growth factors and damage-associated molecular signals that recruit and reprogramme these cellular populations. In turn, infiltrating immune and stromal cells produce mediators that can support tumour-cell survival and proliferation. The intensity and composition of this response may vary according to histological subtype, molecular phenotype, tumour burden and treatment status, contributing to heterogeneity in circulating inflammatory biomarker profiles (18).

Interleukin-6 (IL-6) is a particularly important mediator linking local ovarian tumour inflammation with systemic responses. Ovarian cancer cells, macrophages, fibroblasts and mesothelial cells can produce IL-6. Binding of IL-6 to its receptor activates Janus kinase (JAK) signalling and subsequent signal transducer and activator of transcription 3 (STAT3) phosphorylation. Persistent JAK/STAT3 activation can promote tumour-cell proliferation, resistance to apoptosis, angiogenesis, stem-cell-like phenotypes and immune suppression. IL-6 also stimulates hepatic acute-phase responses, providing a mechanistic link between local tumour inflammation and circulating markers such as C-reactive protein (CRP) and fibrinogen. Thus, elevated circulating IL-6 may reflect more than the presence of inflammation; it may indicate an active tumour–host signalling axis associated with aggressive disease. Nevertheless, IL-6 is not cancer-specific and can increase substantially during infection and other inflammatory states. Interleukin-8 (IL-8; CXCL8) connects inflammatory signalling with neutrophil recruitment, angiogenesis and tumour-cell migration. Ovarian cancer cells and tumour-associated stromal cells can produce IL-8, which acts through CXCR1 and CXCR2 receptors on tumour and immune cells. Increased IL-8 signalling may promote endothelial activation and angiogenesis while facilitating tumour-cell motility and invasion. Recruitment and functional reprogramming of neutrophils can further amplify this process. Tumour-associated neutrophils may release proteases, reactive oxygen species, cytokines and neutrophil extracellular traps (NETs), potentially supporting tumour-cell survival and metastatic dissemination. This biology provides a mechanistic basis for the association between elevated circulating IL-8, neutrophilia and adverse ovarian cancer phenotypes. However, the translation of this mechanism into a clinically reliable blood test remains limited by assay variability and the nonspecific nature of inflammatory activation (19–22).

Tumour necrosis factor-α (TNF-α) is another important inflammatory mediator implicated in ovarian cancer progression. Sustained TNF-α signalling can activate nuclear factor-κB (NF-κB), a transcriptional regulator controlling numerous genes involved in inflammation, cell survival, proliferation, angiogenesis and immune modulation. The TNF-α/NF-κB axis can therefore create a positive feedback loop in which inflammatory signalling promotes tumour-cell survival while tumour and stromal cells continue to generate additional inflammatory mediators. Nevertheless, TNF-α has context-dependent biological effects, and circulating concentrations do not necessarily reflect signalling activity within the tumour microenvironment. This limits its utility as an isolated clinical biomarker (23). Inflammation in ovarian cancer is closely interconnected with haemostatic activation. Tumour cells can activate platelets through direct interactions and soluble mediators, while activated platelets release growth factors, cytokines and angiogenic molecules. Platelets may facilitate tumour progression by protecting circulating tumour cells, promoting endothelial adhesion, supporting angiogenesis and enhancing metastatic implantation. Fibrinogen and other coagulation proteins can further contribute to formation of a pro-tumour microenvironment. This interaction explains the biological relevance of platelet-derived inflammatory indices such as the platelet-to-lymphocyte ratio (PLR), systemic immune-inflammation index (SII) and pan-immune-inflammation value (PIV). These indices may indirectly capture the interaction between thrombosis-associated tumour biology and host immune status (24, 25).

Circulating monocytes can migrate into tumour tissue and differentiate into tumour-associated macrophages (TAMs). Depending on their functional phenotype and local signals, TAMs can promote angiogenesis, extracellular-matrix remodelling, invasion and immune suppression. Ovarian cancer-associated macrophages may produce IL-6, IL-10, transforming growth factor-β and other mediators that influence tumour progression and suppress effective antitumour immune responses. Consequently, an increased monocyte burden combined with reduced lymphocyte representation may indicate an inflammatory environment favourable to tumour progression. This provides the rationale for the lymphocyte-to-monocyte ratio (LMR) as a readily accessible systemic marker. However, circulating monocyte counts are an indirect measure of tumour-associated macrophage activity and should not be interpreted as a direct surrogate for the tumour microenvironment (26). Effective lymphocyte-mediated immunity is an important component of tumour surveillance. Ovarian cancers with active antitumour immune responses may demonstrate greater lymphocytic infiltration, whereas advanced disease can be associated with systemic immune dysfunction and lymphopenia. A reduction in circulating lymphocytes may therefore indicate impaired adaptive immune competence. When combined with neutrophilia or thrombocytosis, lymphopenia produces elevated NLR, PLR, SII or PIV values. The systemic inflammatory response can activate hepatic acute-phase protein synthesis, principally through IL-6-mediated signalling. CRP is consequently an accessible downstream indicator of systemic inflammatory activity. Elevated CRP in ovarian cancer may reflect tumour burden, necrosis, cytokine activity, infection or other inflammatory processes. Its principal clinical advantage is analytical accessibility and reproducibility, while its major limitation is poor disease specificity. Accordingly, CRP should be interpreted as a marker of systemic inflammatory burden rather than a tumour-specific ovarian cancer marker (27, 28).

## Inflammation and ovarian tumorigenesis

Chronic inflammation contributes to ovarian tumorigenesis by creating a microenvironment that favours malignant transformation, cellular survival and tumour progression. Repeated inflammatory stimulation within the ovarian and peritoneal microenvironment can generate reactive oxygen species, cytokines and growth factors that promote DNA damage, genomic instability and aberrant cellular proliferation. Cytokines such as interleukin-6 (IL-6), interleukin-8 (IL-8) and tumour necrosis factor-α (TNF-α) activate signalling pathways including JAK/STAT3 and nuclear factor-κB (NF-κB), supporting proliferation, resistance to apoptosis and immune evasion (17, 18). Inflammation also remodels the tumour microenvironment through recruitment of macrophages, neutrophils, fibroblasts and other stromal cells. These populations can release angiogenic and matrix-remodelling mediators that facilitate tumour expansion and invasion. Persistent inflammatory signalling may additionally promote epithelial–mesenchymal transition, enhancing cellular motility and metastatic potential (20). Inflammation in ovarian cancer is bidirectional: malignant cells stimulate inflammatory responses, while inflammatory cells and mediators can reciprocally promote tumour progression. This tumour–inflammation feedback loop provides the biological basis for circulating inflammatory biomarkers. However, because systemic inflammation also occurs in numerous non-malignant conditions, its presence alone cannot distinguish ovarian cancer from other inflammatory states. Thus, inflammatory biomarkers are most appropriately viewed as complementary indicators of tumour–host biology rather than standalone markers of tumorigenesis (21,22).

## Blood-based inflammatory biomarkers

Blood-based inflammatory biomarkers provide minimally invasive measures of the systemic host response associated with ovarian cancer. They broadly capture three interconnected biological domains: circulating inflammatory mediators, acute-phase/coagulation proteins, and peripheral blood-cell profiles. Their major advantage is accessibility, low cost and compatibility with routine laboratory testing; however, their lack of cancer specificity remains a major barrier to clinical implementation. C-reactive protein (CRP) is an acute-phase protein induced predominantly by IL-6-mediated hepatic signalling. Increased CRP has been associated with advanced ovarian cancer, greater tumour burden and poorer survival. Fibrinogen similarly links inflammation with coagulation and may facilitate platelet activation, tumour-cell adhesion, angiogenesis and metastatic implantation. Elevated concentrations of both markers may therefore reflect an aggressive tumour–host phenotype. Nevertheless, infection, tissue injury, autoimmune disease and other inflammatory conditions can substantially increase their concentrations, limiting their diagnostic specificity (28).

IL-6, IL-8 and TNF-α provide more direct insight into inflammatory signalling. IL-6 promotes JAK/STAT3 activation, tumour-cell survival and immune dysregulation, whereas IL-8 contributes to neutrophil recruitment, angiogenesis and tumour-cell migration. TNF-α can activate NF-κB-dependent inflammatory and survival pathways. Elevated circulating concentrations have been associated with advanced disease and adverse outcomes in several studies. However, cytokine concentrations are highly dynamic and influenced by infection, treatment, sampling conditions and assay methodology, restricting their use as standalone clinical biomarkers (29). The neutrophil-to-lymphocyte ratio (NLR) combines tumour-associated innate inflammatory activation with adaptive immune competence. Neutrophilia may promote tumour progression through cytokine release, proteolytic activity and neutrophil extracellular traps, whereas lymphopenia may indicate impaired antitumour immunity. Elevated NLR is frequently associated with advanced disease and poorer survival. Its simplicity and availability are major strengths, but infection, corticosteroids and other inflammatory conditions can markedly alter the ratio (33).

The platelet-to-lymphocyte ratio (PLR) reflects the interaction between thrombocytosis and lymphocyte-mediated immunity. Activated platelets can protect circulating tumour cells, promote endothelial adhesion and release proangiogenic mediators. Consequently, elevated PLR has been associated with aggressive disease and unfavourable outcomes. However, PLR remains nonspecific and is influenced by numerous non-malignant and treatment-related factors (30). The lymphocyte-to-monocyte ratio (LMR) provides an indirect estimate of adaptive immune competence relative to myeloid inflammatory activity. Monocytes can differentiate into tumour-associated macrophages that promote angiogenesis, extracellular-matrix remodelling and immunosuppression. A lower LMR has therefore been associated with adverse ovarian cancer characteristics in several studies, although its clinical thresholds and independent prognostic value remain inconsistent (31). Composite indices—including the systemic immune-inflammation index (SII), systemic inflammation response index (SIRI) and pan-immune-inflammation value (PIV)—integrate multiple circulating cell populations. SII incorporates platelets, neutrophils and lymphocytes; SIRI incorporates neutrophils, monocytes and lymphocytes; and PIV integrates neutrophils, monocytes, lymphocytes and platelets. Their principal advantage is broader representation of the inflammatory–immune state than individual ratios. However, greater biological complexity has not consistently translated into superior clinical performance. Variable cut-offs, retrospective study designs and limited prospective validation remain important limitations (Table 1) (32).

**TABLE 1:** Blood-based inflammatory biomarkers in ovarian cancer: biological functions, clinical significance, and limitations.

Biomarker	Biological function	Clinical significance in ovarian cancer	Key limitations
CRP	Reflects IL-6-driven hepatic acute-phase response	Associated with tumour burden, advanced disease and poorer survival	Low cancer specificity; affected by infection and systemic inflammation
IL-6	Activates JAK/STAT3 signalling, promoting survival, proliferation and immune dysregulation	Associated with aggressive disease, progression and possible treatment resistance	Dynamic concentrations; assay and biological variability
IL-8 (CXCL8)	Promotes neutrophil recruitment, angiogenesis and tumour-cell migration	Potential indicator of invasion, angiogenesis and metastatic activity	Limited standardisation; influenced by non-malignant inflammation
TNF-α	Activates NF-κB and inflammatory survival pathways	May support tumour progression and immune modulation	Context-dependent effects; poor standalone specificity
Fibrinogen	Couples inflammation with coagulation and platelet activation	Associated with tumour progression, metastasis and adverse prognosis	Increased in infection, tissue injury and other inflammatory states
NLR	Integrates neutrophil-mediated inflammation with lymphocyte-mediated immunity	Frequently associated with advanced disease and reduced survival	Cut-off heterogeneity; affected by infection, corticosteroids and comorbidities
PLR	Reflects platelet activation relative to lymphocyte abundance	Potential marker of tumour-associated inflammation and adverse prognosis	Nonspecific; influenced by thrombosis, inflammation and treatment
LMR	Balances lymphocyte-mediated immunity against monocyte/myeloid activity	Low values may indicate an immunosuppressive tumour-associated phenotype	Indirect surrogate of tumour immunity; inconsistent thresholds
SII	Integrates platelets, neutrophils and lymphocytes	Potentially improves systemic inflammatory risk stratification; may assist treatment-selection assessment	Variable cut-offs; limited prospective validation
SIRI	Integrates neutrophils, monocytes and lymphocytes	May reflect combined innate, myeloid and adaptive immune dysregulation	Evidence remains predominantly observational
PIV	Integrates neutrophils, monocytes, platelets and lymphocytes	Provides a broader measure of systemic inflammatory burden and potential prognostic risk	Greater complexity without consistently demonstrated superiority; requires validation

Abbreviations: CRP, C-reactive protein; IL, interleukin; JAK/STAT3, Janus kinase/signal transducer and activator of transcription 3; TNF-α, tumour necrosis factor-α; NF-κB, nuclear factor-κB; NLR, neutrophil-to-lymphocyte ratio; PLR, platelet-to-lymphocyte ratio; LMR, lymphocyte-to-monocyte ratio; SII, systemic immune-inflammation index; SIRI, systemic inflammation response index; PIV, pan-immune-inflammation value.

## Inflammatory biomarkers in early detection

Early detection remains the principal unmet need in ovarian cancer, but the current evidence does not support the use of inflammatory biomarkers as standalone screening tests. Markers such as CRP, IL-6, IL-8, NLR, PLR, SII, SIRI and PIV can reflect tumour-associated inflammation and may differ between patients with ovarian cancer and non-malignant controls; however, substantial overlap occurs because these biomarkers are also altered by infection, obesity, autoimmune disease, tissue injury and other inflammatory conditions. Their diagnostic performance is therefore generally insufficient to achieve the specificity required for population screening (25). The potential value of inflammatory biomarkers is greater as complementary components of multimarker strategies. CA-125 remains clinically useful but has limited sensitivity and specificity, particularly in early-stage disease and benign inflammatory conditions, while HE4 provides complementary tumour-associated information. Incorporating inflammatory indices with CA-125, HE4, menopausal status, symptoms and imaging may improve discrimination between women at low and high risk of clinically significant ovarian malignancy (26). Composite indices such as SII, SIRI and PIV may theoretically provide broader biological information because they integrate several components of systemic inflammation and immune competence. Nevertheless, their apparent diagnostic advantages require cautious interpretation because reported cut-offs and performance estimates vary substantially between studies (27, 28). Statistical association does not establish screening utility. Demonstrating that an inflammatory marker is elevated in ovarian cancer is insufficient without prospective validation showing clinically meaningful sensitivity, specificity, calibration and improvement in patient outcomes. Future research should therefore prioritise longitudinal, multicentre studies focused specifically on stage I and II disease, should determine whether inflammatory profiles provide incremental diagnostic value beyond CA-125, HE4, and established clinical and imaging parameters (29, 33).

## Diagnostic utility of inflammatory biomarkers in ovarian cancer

Blood-based inflammatory biomarkers have potential diagnostic value because they provide accessible measures of the systemic response associated with ovarian malignancy. Elevated NLR, PLR, SII, SIRI, PIV, CRP, IL-6 and IL-8 have been reported in patients with ovarian cancer, particularly those with advanced disease. However, their diagnostic utility is substantially weaker than their potential prognostic value because systemic inflammation is not specific to ovarian malignancy (30). Among haematological indices, NLR and SII have received considerable attention because they integrate opposing components of inflammatory activation and adaptive immune competence. Composite indices such as SII, SIRI and PIV may theoretically capture a broader inflammatory phenotype than individual ratios, but evidence that they consistently outperform NLR or established tumour markers remains limited. Differences in study populations, disease stage, inflammatory cut-offs, laboratory methods and statistical approaches contribute to inconsistent diagnostic estimates (31).

Circulating cytokines, particularly IL-6 and IL-8, may provide greater biological insight because they participate directly in tumour-promoting inflammatory pathways. Nevertheless, their short biological half-lives, pre-analytical variability, assay costs and elevation in non-malignant inflammatory conditions restrict immediate clinical application. Similarly, CRP and fibrinogen are readily available but lack adequate specificity for cancer diagnosis (32). Consequently, inflammatory biomarkers should not be interpreted as independent diagnostic tests or population-screening tools. Their most plausible role is as complementary variables within multimarker models combining CA-125, HE4, clinical characteristics and imaging. Such integration may improve risk discrimination while reducing reliance on any single nonspecific biomarker (16). Future diagnostic studies should focus specifically on early-stage disease, use prospectively defined thresholds, include appropriate benign and healthy control groups, and demonstrate incremental diagnostic value beyond established biomarkers (Tables 2, 3) (33).

**TABLE 2:** Prognostic value of NLR and PLR.

Evidence strength	Claim
Evidence strength: Strong (8/10)	Elevated NLR independently predicts worse overall survival (pooled HR 1.70, 95% CI 1.35–2.15) and progression-free survival (HR 1.77, 95% CI 1.48–2.12) in ovarian cancer across 13 retrospective studies enrolling 3,467 patients (35, 36)
Evidence strength: Strong (8/10)	High PLR is associated with poorer OS (pooled HR 2.05, 95% CI 1.70–2.48) and PFS (HR 1.85, 95% CI 1.53–2.25), with a stronger effect than NLR in multiple meta-analyses (36, 37)
Evidence strength: Moderate (5/10)	NLR at a cut-off of 1.93 is an independent predictor of OS (HR 2.23, 95% CI 1.08–4.61) but not PFS in a single-center cohort of 93 EOC patients (38)

**TABLE 3:** Diagnostic performance of inflammatory markers.

Marker/Combination	AUC	Sensitivity	Key finding
SII alone	0.743	71.64%	Diagnostic accuracy nearly comparable to CA125 (sensitivity 75.97%) in 132 patients (39)
CA125+HE4+PLR + MLR + SII	0.951	84.66%	Highest sensitivity among all combinations for distinguishing EOC from benign tumors (40)
Six inflammatory-nutritional markers combined	0.881	78.20%	Composite marker outperformed any individual inflammatory marker alone (41)
CA125+NLR + PLR	—	—	Combined use improved identification of borderline ovarian tumors over any marker alone (42)

## Inflammatory biomarkers and metastasis

Metastatic dissemination is a defining feature of advanced ovarian cancer and occurs predominantly through transcoelomic spread within the peritoneal cavity. Inflammation can facilitate this process by promoting tumour-cell survival, epithelial–mesenchymal transition (EMT), extracellular-matrix remodelling, angiogenesis and adhesion to peritoneal surfaces. However, circulating inflammatory biomarkers are indirect measures of these processes and should not be interpreted as definitive evidence of metastatic disease. Neutrophils may promote metastatic progression through secretion of proteases, reactive oxygen species, cytokines and neutrophil extracellular traps (NETs), which can enhance tumour-cell migration, vascular or mesothelial adhesion and immune evasion. Consequently, an elevated NLR has frequently been associated with advanced stage, greater tumour burden, ascites and poorer outcomes. Nevertheless, NLR cannot reliably distinguish metastatic ovarian cancer from other systemic inflammatory states and should therefore complement, rather than replace, imaging and surgical assessment (41).

Platelets interact directly with circulating tumour cells and may facilitate their protection from immune-mediated destruction, adhesion to endothelium or mesothelium and subsequent metastatic implantation. Fibrinogen further links coagulation with inflammation by supporting platelet–tumour interactions and extracellular matrix formation. Elevated PLR and fibrinogen have consequently been associated in several studies with aggressive disease and adverse outcomes. Their clinical interpretation remains limited by substantial effects of thrombosis, infection, tissue injury and other inflammatory conditions (42). Circulating monocytes can migrate into the tumour microenvironment and differentiate into tumour-associated macrophages, which may promote angiogenesis, matrix remodelling, invasion and immunosuppression. A reduced LMR may therefore reflect a systemic phenotype characterised by increased myeloid activity and impaired lymphocyte-mediated surveillance. However, circulating monocyte counts are only indirect surrogates of tumour-associated macrophage function, limiting the specificity of LMR for metastatic biology (27). SII, SIRI and PIV integrate multiple circulating cell populations and may capture the combined inflammatory and immune dysregulation associated with metastatic progression. Higher values have been linked in observational studies to advanced disease and poorer survival. Their principal advantage is broader biological representation, whereas their major limitations include variable thresholds, dependence on routine blood-count parameters and uncertainty regarding whether they provide clinically meaningful information beyond stage, tumour burden, CA-125 and imaging (43).

## Prognostic significance in metastasis and survival in ovarian cancer

Inflammation-driven pathways are intricately linked to the metastatic potential and clinical outcomes of ovarian cancer. Systemic inflammatory responses, as reflected by peripheral blood biomarkers, have gained recognition not only for their diagnostic relevance but also for their ability to stratify patients by prognosis. These markers offer a real-time, cost-effective means of gauging tumor aggressiveness, likelihood of metastasis, and survival trajectories, thereby supporting clinical decision-making in both initial and recurrent disease settings (34). Among the most studied prognostic indicators are the neutrophil-to-lymphocyte ratio (NLR), platelet-to-lymphocyte ratio (PLR), and systemic immune-inflammation index (SII). Elevated pre-treatment NLR and PLR levels have consistently been associated with reduced progression-free survival (PFS) and overall survival (OS) in ovarian cancer patients. High NLR values, for instance, reflect a dominance of neutrophil-mediated pro-tumor inflammation over lymphocyte-mediated immune surveillance, correlating with chemoresistance, rapid disease progression, and distant metastasis. Similarly, a high PLR often reflects platelet-facilitated tumor cell migration and protection from immune attack, both of which are key drivers of metastatic spread (44).

The systemic immune-inflammation index (SII), which integrates neutrophil, lymphocyte, and platelet counts, has shown even stronger prognostic utility than NLR or PLR alone. Elevated SII has been linked to increased peritoneal dissemination, higher tumor burden, and poor response to cytoreductive surgery and chemotherapy. Importantly, patients with lower SII values tend to exhibit better surgical outcomes, reduced risk of recurrence, and longer survival, underscoring the role of inflammatory burden in shaping clinical trajectories (45). C-reactive protein (CRP), another widely studied marker, is a strong predictor of poor survival outcomes. Elevated CRP levels reflect ongoing tumor-related inflammation and have been correlated with advanced-stage disease, extensive ascites, and reduced treatment efficacy. Persistently high CRP during or after chemotherapy may serve as an early indicator of subclinical disease progression or recurrence. Its dynamic changes over time can thus guide treatment monitoring and re-evaluation of therapeutic strategies (46).

Cytokines such as interleukin-6 (IL-6) and interleukin-8 (IL-8) further reinforce the prognostic significance of inflammation in ovarian cancer. High serum IL-6 levels are associated with advanced-stage disease, platinum resistance, and reduced OS, largely due to their roles in promoting angiogenesis, immunosuppression, and tumor growth. IL-8 enhances metastatic potential by facilitating epithelial-to-mesenchymal transition (EMT) and enabling tumor cell motility and invasion. These cytokines may also contribute to cancer cachexia and systemic immune dysfunction, further worsening patient prognosis (38). Incorporating inflammatory biomarkers into prognostic models has shown potential to outperform conventional clinical parameters such as FIGO stage or residual disease alone. Composite indices that combine inflammatory markers with tumor burden, imaging features, and molecular signatures may offer more robust survival predictions and help identify high-risk patients who could benefit from intensified treatment or immunomodulatory therapies (47).

## Clinical implications in ovarian cancer

The clinical utility of blood-based inflammatory biomarkers in ovarian cancer extends beyond diagnosis and prognosis, offering tangible benefits in patient stratification, therapeutic planning, and treatment monitoring. These biomarkers serve as dynamic indicators of the tumor-host interplay, allowing clinicians to gauge disease aggressiveness, predict treatment response, and detect recurrence earlier than traditional imaging or symptom-based approaches. Their integration into routine clinical workflows could revolutionize how ovarian cancer is managed, particularly in resource-limited settings where access to advanced imaging or molecular profiling is constrained (48). In preoperative settings, inflammatory indices such as the neutrophil-to-lymphocyte ratio (NLR), platelet-to-lymphocyte ratio (PLR), and systemic immune-inflammation index (SII) can help guide surgical decisions. Patients with low inflammatory burden are more likely to achieve optimal cytoreduction and better post-operative outcomes. Conversely, elevated markers may suggest an aggressive tumor phenotype and the need for neoadjuvant chemotherapy. These insights can enhance individualized care plans and minimize unnecessary surgical morbidity in patients unlikely to benefit from immediate debulking (49).

During chemotherapy, inflammatory biomarkers offer a means to monitor treatment efficacy and anticipate resistance. Persistent or rising levels of markers such as C-reactive protein (CRP), NLR, or IL-6 may indicate poor response, prompting early reassessment of the therapeutic regimen. Additionally, these biomarkers could serve as surrogate endpoints in clinical trials, allowing for more timely evaluation of novel anti-inflammatory or immunomodulatory agents. Inflammatory markers also provide prognostic information post-treatment, helping clinicians tailor surveillance intervals and supportive care strategies based on recurrence risk (50). From a therapeutic standpoint, targeting inflammation itself represents a novel frontier in ovarian cancer management. Anti-inflammatory agents, cytokine inhibitors, and immunomodulators are being explored as adjuncts to conventional therapies, aiming to disrupt the pro-tumorigenic inflammatory microenvironment. For instance, IL-6 blockade has demonstrated potential in reversing chemoresistance and improving immune responsiveness. Patients with elevated baseline inflammatory markers may particularly benefit from such approaches, highlighting the role of these biomarkers in patient selection for personalized therapies (51). Furthermore, the incorporation of inflammatory biomarkers into composite scoring systems—alongside clinical variables and traditional tumor markers like CA-125—can improve risk stratification and prognostication. These multi-dimensional models have the potential to enhance shared decision-making, optimize resource allocation, and inform eligibility for clinical trials. In the era of precision medicine, such integrative frameworks are essential for transitioning from reactive to proactive cancer care (43, 44).

## Future research directions and practical clinical recommendations

Future research should move beyond retrospective association studies towards prospective, multicentre validation of inflammatory biomarkers across diverse ovarian cancer populations. Studies should prioritise early-stage disease and include appropriate healthy and benign gynaecological controls to establish whether inflammatory biomarkers provide diagnostic information beyond CA-125 and HE4. Standardised blood-sampling protocols, laboratory methods and predefined cut-offs are essential to improve reproducibility and facilitate comparison across studies. Comparative studies should directly evaluate NLR, PLR, LMR, SII, SIRI and PIV rather than assuming that greater mathematical complexity provides superior clinical performance. Longitudinal sampling before treatment, during therapy and at recurrence could determine whether changes in inflammatory profiles provide clinically meaningful information regarding treatment response and disease progression. Importantly, future prediction models should assess incremental value beyond FIGO stage, histological subtype, CA-125, HE4, imaging and established molecular biomarkers using discrimination, calibration and decision-curve analyses. Research should also clarify whether inflammatory biomarkers can contribute to treatment selection. The reported association of preoperative NLR and SII with selection of neoadjuvant chemotherapy highlights this possibility, but prospective studies are required to determine whether biomarker-guided decisions improve outcomes rather than merely reflect existing clinical selection practices. Integration with molecular and liquid-biopsy approaches may ultimately provide more robust biological phenotyping than inflammatory indices alone (39–41).

At present, blood-based inflammatory biomarkers should not be used as standalone screening or diagnostic tests for ovarian cancer. Their nonspecific nature and variability in thresholds preclude replacement of CA-125, HE4, imaging or histopathological assessment. In routine practice, NLR, PLR, SII, SIRI, PIV, CRP and fibrinogen may be considered adjunctive markers of systemic inflammatory burden and risk, particularly when interpreted alongside tumour stage, disease burden, clinical status and established biomarkers. Abnormal results should be interpreted cautiously after considering infection, autoimmune disease, obesity, medications, recent surgery, thrombosis and other potential confounders. Where inflammatory indices are incorporated into clinical prediction models, laboratories and clinicians should use predefined and locally validated thresholds rather than applying arbitrary cut-offs derived from individual retrospective studies. Repeated measurements may be more informative than a single value when monitoring treatment or disease trajectory (27, 42, 43).

## Conclusion

Blood-based inflammatory biomarkers provide biologically plausible and clinically accessible measures of the host response to ovarian cancer, with growing evidence linking elevated CRP, cytokines, NLR, PLR, SII, SIRI and PIV to advanced disease, metastatic burden and adverse outcomes. However, their clinical significance should be interpreted cautiously. The current evidence is predominantly observational and is limited by heterogeneous populations, inconsistent biomarker thresholds, differences in sampling and laboratory methods, retrospective study designs, and confounding by infection, comorbidities, treatment and other non-malignant inflammatory conditions. Current evidence does not support inflammatory biomarkers as standalone tools for population screening or early diagnosis of ovarian cancer. Their diagnostic performance in early-stage disease remains insufficiently established, and prognostic associations should not be interpreted as evidence of treatment-predictive utility. Although composite indices such as SII, SIRI and PIV may capture broader inflammatory phenotypes than individual ratios, their superiority over simpler markers has not been consistently demonstrated. The most credible clinical opportunity is therefore multimodal integration of inflammatory biomarkers with CA-125, HE4, imaging, FIGO stage, histological characteristics and molecular biomarkers. Such integration may improve risk stratification and potentially inform treatment selection, but prospective evidence demonstrating improved clinical outcomes remains necessary. Future research should prioritise multicentre prospective validation, standardised thresholds, longitudinal measurements, external validation and decision-analytic assessment of clinical utility. Until such evidence is available, inflammatory biomarkers should be regarded as complementary indicators of tumour–host biology rather than established standalone diagnostic or prognostic tests.
